# Blockade of CD354 (TREM-1) Ameliorates Anti-GBM-Induced Nephritis

**DOI:** 10.1007/s10753-016-0351-1

**Published:** 2016-04-15

**Authors:** Yong Du, Tianfu Wu, Xin J. Zhou, Laurie S. Davis, Chandra Mohan

**Affiliations:** Division of Rheumatology, Department of Internal Medicine, University of Texas Southwestern Medical Center, 5323 Harry Hines Blvd, Bldg Y, Flr 8, Room 206 (Y8.206), Dallas, TX 75390-8884 USA; Department of Biomedical Engineering, University of Houston, 3605 Cullen Blvd, Room 2027, Houston, TX 77204-5060 USA; Department of Pathology, University of Texas Southwestern Medical Center, Dallas, TX 75390 USA

**Keywords:** anti-glomerular basement membrane antibody-induced nephritis, Triggering Receptor Expressed by Myeloid Cells-1 (TREM-1), immune-mediated nephritis, SLE, systemic lupus erythematosus, immunotherapy

## Abstract

CD354, Triggering Receptor of Myeloid Cells-1 (TREM-1), is a potent amplifier of myeloid immune responses. Our goal was to determine the expression and function of TREM-1 in immune-mediated nephritis. An anti-glomerular basement membrane antibody (anti-GBM)-induced nephritis model was employed, where mice were sensitized with rabbit IgG followed by anti-GBM serum to induce disease. Anti-GBM-treated 129x1/svJ mice developed severe nephritis whereas C57BL/6 (B6) mice were resistant to disease. Anti-GBM disease resulted in elevated renal TREM-1 messenger RNA (mRNA) and protein levels and increased urine TREM-1 levels in 129x1/svJ. TREM-1 blockade with an inhibitory peptide, LP17, inhibited proteinuria and renal disease as measured by glomerulonephritis class, severity of tubulointerstitial disease, crescent formation, and inflammatory cell infiltrates. In sum, TREM-1 is upregulated in renal inflammation and plays a vital role in driving disease. Thus, TREM-1 blockade emerges as a potential therapeutic avenue for immune-mediated renal diseases such as lupus nephritis.

## INTRODUCTION

Renal inflammation leading to organ dysfunction and end-stage disease is a common feature of autoimmune diseases such as systemic lupus erythematosus (SLE) [1, 2]. Increased knowledge of pathogenic mechanisms could greatly enhance prognosis and treatment of kidney diseases. Murine models suggest that contributing factors to immune-mediated nephritis include autoantibodies such as anti-glomerular antibodies [3, 4] and innate immune activation independent of autoantibody production [5, 6]. Dendritic cells maintain renal self-tolerance mediated by T regulatory cells [7]. However, resident macrophages and recruited monocytes regulate renal inflammation which can involve renal cells [8, 9].

Anti-glomerular basement membrane antibody (anti-GBM)-induced disease or Goodpasture’s syndrome is characterized by antibodies with specificities to renal targets, such as collagen type IV in glomerular basement membranes [10]. Murine models mimicking human anti-GBM nephritis require immunization with collagen type IV or heterologous anti-GBM antibodies which initiate an autoimmune antibody-mediated inflammatory response [10]. The murine anti-GBM model shares a number of characteristics with spontaneous lupus murine models in regard to pathogenic mechanisms contributing to nephritis including shared molecular mediators such as complement, FcR, toll-like receptors (TLR), costimulatory receptors, adhesion receptors, cytokines, chemokines, and other mediators [11–13]. Thus, the anti-GBM model provides a unique approach for rapidly screening agents with potential therapeutic efficacy for other more protracted varieties of immune-mediated nephritis such as lupus nephritis.

This study was designed to determine whether the novel inhibitory peptide LP17, targeting the CD354 receptor, Triggering Receptor Expressed on Myeloid Cells-1 or TREM-1, could impact anti-GBM nephritis. TREM-1 was first described as a costimulatory receptor on myeloid cells [14, 15]. TREM-1 potently amplified the function of TLR and Nod-like receptors (NLR) which resulted in enhanced cytokine production [14, 15]. Early studies reported a role for TREM-1 in sepsis models and found that soluble TREM-1 could serve as a biomarker for acute inflammation during infection [16, 17]. Protective effects of TREM-1 blockade were also documented in acute infections [14, 18]. Subsequently, a role for TREM-1 in the amplification of noninfectious inflammatory responses was reported [14, 17, 19]. Recently, TREM-1 activation by self-proteins in the absence of TLR/NLRs has been shown to stimulate monocyte cytokine production [20]. Antagonizing TREM-1 with antibodies, fusion proteins, or inhibitory peptides reduces inflammation in experimental arthritis and colitis models [14, 21, 22]. This report represents a first step toward revealing the contribution of TREM-1 to inflammatory kidney diseases. The current study demonstrates that the LP17 inhibitory peptide targeting TREM-1 profoundly subdues anti-GBM-mediated nephritis.

## MATERIALS AND METHODS

### Mice and Anti-GBM Nephritis Model

Female 129x1/svJ mice and C57BL/6 (B6) controls (Jackson Laboratories) were maintained in a pathogen-free colony. Animal experiments were approved and conducted in accordance with UT Southwestern’s Institutional Animal Care and Use Committee guidelines which comply with all applicable international and national guidelines for the care and use of animals. Anti-GBM serum was purchased from Lampire Laboratories [23, 24]. Anti-GBM nephritis was induced in 8–12-week-old female mice [4, 23]. Briefly, mice were pre-sensitized with rabbit IgG 250 μg/mouse, given intraperitoneally (i.p.) in complete Freund’s adjuvant (Sigma). Five days later, the mice received rabbit anti-GBM serum 200 μg IgG/25 g body weight, administered intravenously (i.v.) [4, 23]. This dosing regimen was sufficient to induce proteinuria but not mortality. Experiments are representative of three independent experiments.

### TREM-1 Blockade

Upon nephritis induction, mice were randomly assigned to receive either an antagonistic TREM-1 peptide, LP17 (LQVTDSGLYRCVIYHPP), a sequence-scrambled control peptide (TDSRCVIGLYHPPLQVY), or 100 μl phosphate buffered saline (PBS) alone as vehicle control [18]. The peptides were chemically synthesized as COOH terminally amidated peptides (Pepscan Systems). Mice were treated daily with 200 μg peptide in 50–100 μl PBS through i.p. injection initiated on day 7 (D7). Weekly serum samples and 24-h urine samples were collected as described [4, 23, 24].

### Renal Histopathology

Kidney specimens were prepared as 4-μm sections of formalin-fixed, dehydrated, and paraffin-embedded tissues [4, 23, 24]. Slides were stained with hematoxylin and eosin or periodic acid-Schiff (PAS). Replicate sections were examined for pathological changes in glomeruli, tubules, or interstitial areas in a blinded fashion [4, 25]. Glomerulonephritis (GN) was graded on a 0–5 scale, while percent crescent formation and the tubulointerstitial nephritis (TI) score were graded on 0–4 scales [4, 23, 24]. Macrophages were stained with Iba1 (Wako Chemicals), and intrarenal leukocytes and macrophages were enumerated. Standard immunofluorescence and avidin-biotin complex methods were used for target detection [4, 25].

### Sample Preparation and TREM-1 Enzyme-Linked Immunosorbent Assay (ELISA)

All serum and urine samples were collected on the days indicated and centrifuged at 14,000 rpm for 5 min, immediately aliquoted and rapidly frozen. Clear supernatants were assayed for total protein with a Coomassie Plus Kit (Pierce). The serum and urine creatinine levels were measured by capillary electrophoresis diode array detector in the UT Southwestern Metabolomics core. Proteinuria was detected for samples collected over a 24-h period as described [25]. Renal eluates were prepared as described [4, 23, 24]. Soluble TREM-1 levels were measured by ELISA (R&D Systems).

### Illumina Arrays and RT-PCR Analysis

Renal macrophages isolated by Miltenyi CD11b magnetic selection were 95 % purified populations [26]. Total RNA was purified using RNeasy Kits (Qiagen) and the RNA quality was checked with an Agilent Bioanalyzer (Agilent Technologies). Microarray analysis was performed using the Sentrix Mouse-6 v1.1 Illumina Whole Genome Expression Beadchips according to the manufacturer’s protocol. Microarray data using background noise subtracted and normalized data (BeadStudio v3.1 software) was assessed as previously reported [27]. Data is shown as the average signal intensity for TREM-1 and TREM-2 messenger RNA (mRNA) levels.

Renal cortex and medullas were isolated under a dissecting microscope (Zeiss) and total cellular RNA was prepared as previously described [25]. Quantitative polymerase chain reaction (Q-PCR) was performed to detect TREM-1 mRNA expression in kidney tissue using the TaqMan reverse transcription reagent (Applied Biosystems). Primers were as follows: mouse TREM-1, forward 5′-GAGCTTGAAGGATGAGGAAGGC-3′ and reverse 5′-CAGAGTCTGTCACTTGAAGGTCAGTC-3′; mouse β-actin, forward 5′-TGGAATCCTGTGGCATCCATGAAAC-3′ and reverse 5′-TAAAACGCAGCTCAGTAACAGTCCG-3. Expression of TREM-1 was normalized to β-actin expression using the standard ΔCT method.

### Statistical Analysis

Data analysis was performed using the Mann-Whitney *U* test or linear regression as described in the text with either SigmaStat (SPSS, Chicago, IL) or GraphPad Prism software (San Diego, CA).

## RESULTS

### Enhanced TREM-1 Expression in Anti-GBM-Induced Nephritis in 129/SvJ Mice

**Fig. 1:**
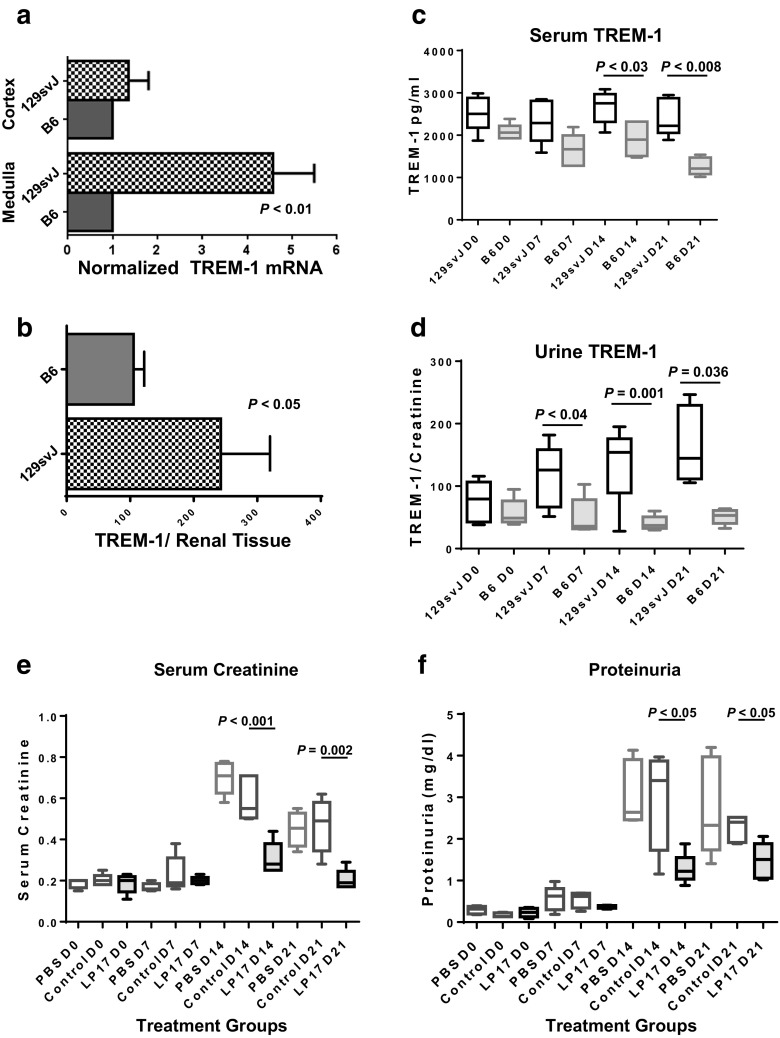
Elevated TREM-1 mRNA and protein expression in anti-GBM-induced nephritis. Anti-GBM was induced and 7 days later, the kidneys were examined for TREM-1 mRNA and protein. **a** TREM-1 mRNA levels were elevated in the renal medulla of 129/SvJ compared to B6 mice. **b** TREM-1 protein was detected by ELISA in renal eluates from 129/SvJ and control B6 mice. **c** Soluble TREM-1 levels were reduced in the serum of control B6 mice compared to the 129/SvJ mice on days 14 and 21 as a result of an actual decrease in soluble TREM-1 levels in B6 mice over the course of disease. **d** Soluble TREM-1 was significantly elevated in the urine of 129/SvJ compared to control B6 mice. Soluble TREM-1 levels were also elevated in the urine of 129/SvJ on day 21 compared to 129/SvJ on day 0 (*P* < 0.05). Urine soluble TREM-1 levels were normalized to creatinine. TREM-1 blockade inhibits anti-GBM disease in 129/SvJ mice. Anti-GBM-induced mice were randomly divided into three groups (*n* = 5 each) receiving (1) PBS, (2) control scrambled peptide, or (3) LP17 peptide. Mice received daily treatment with controls or LP17 peptide. **e** Serum creatinine levels were elevated on days 14–21 in mice receiving PBS or control peptide, but only slightly elevated in LP17-treated mice. **f** Proteinuria was observed in control mice, but not in LP17-treated mice through day 21. Statistical analysis was carried out by Mann-Whitney *U* tests to obtain the *P* values shown.

We have previously shown that the 129/SvJ strain is susceptible to a rapid onset glomerulonephritis while C57BL/6 (B6) mice are relatively resistant to anti-GBM nephritis [4, 23, 25, 28]. TREM-1 and TREM-2 were undetectable in control kidneys by immunohistochemistry (IHC) before induction of anti-GBM disease. We examined the ratio of TREM-1 to TREM-2 mRNA expression by macrophages in anti-GBM-diseased kidneys on day 7 by Illumina arrays. An increased TREM-1/TREM-2 ratio [TREM-1, 228.2 ± 18.6 (mean ± SD), TREM-2 931.2 ± 184.2, (*n* = 3 each group)] was observed in 129/SvJ kidneys as compared to control B6 kidneys [TREM-1, 65.74 ± 9.06, TREM-2 4914 ± 566.9]. B6 macrophages displayed markedly increased TREM-2 expression. Of note, TREM-2 has been associated with immunosuppressive myeloid cells. Thus, this finding is consistent with the resistance of the B6 strain to anti-GBM nephritis [15, 29, 30]. To confirm the increased TREM-1 mRNA in 129/SvJ anti-GBM-induced kidneys, we carried out real-time Q-PCR (Fig. 1a). Elevations in TREM-1 mRNA levels were found in the renal medulla of 129/SvJ mice, while cortical TREM-1 levels were similar in both strains. We next examined TREM-1 protein levels. Renal eluates from anti-GBM-induced 129/SvJ mice contained increased soluble TREM-1 protein levels as compared to B6 controls (Fig. 1b). Although soluble TREM-1 was elevated in the serum of 129/SvJ compared to control B6 mice at later timepoints (days 14 and 21), this was correlated with a slight decrease in B6 serum soluble TREM-1 levels at days 14 and 21 in response to anti-GBM treatment (Fig. 1c). There was no significant difference between 129/SvJ serum soluble TREM-1 levels on day 0 compared to days 7, 14, and 21. (Fig. 1c). Soluble TREM-1 levels normalized to urine creatinine were also significantly elevated in anti-GBM-induced 129/SvJ urine on days 7, 14, and 21 as compared to B6 controls (Fig. 1d). Moreover, soluble TREM-1 levels were also elevated in the urine of 129/SvJ on day 21 compared to 129/SvJ on day 0 (*P* < 0.05). These studies confirmed that TREM-1 mRNA and protein were both elevated in anti-GBM 129/SvJ nephritis compared to the B6 controls. Thus, increased renal TREM-1 levels correlated with disease severity.

### TREM-1 Blockade Ameliorates Renal Injury

To determine whether TREM-1 played a pathogenic role in anti-GBM-induced nephritis, we treated 129/SvJ mice with an antagonistic TREM-1 peptide, LP17 [18, 31]. Renal function was monitored by assessing serum creatinine levels and urine protein excretion (Fig. 1e, f). Serum creatinine levels were elevated on days 14 and 21 in vehicle and peptide control-treated animals whereas minimal changes were observed in LP17-treated mice (Fig. 1e). Proteinuria was markedly increased in control animals on days 14 and 21, but not in samples from LP17-treated mice (Fig. 1f). These studies suggest that anti-GBM-induced renal impairment was significantly improved by treatment with the LP17 peptide as compared to controls.

### Renal Pathology in LP17-Treated Mice

**Fig. 2:**
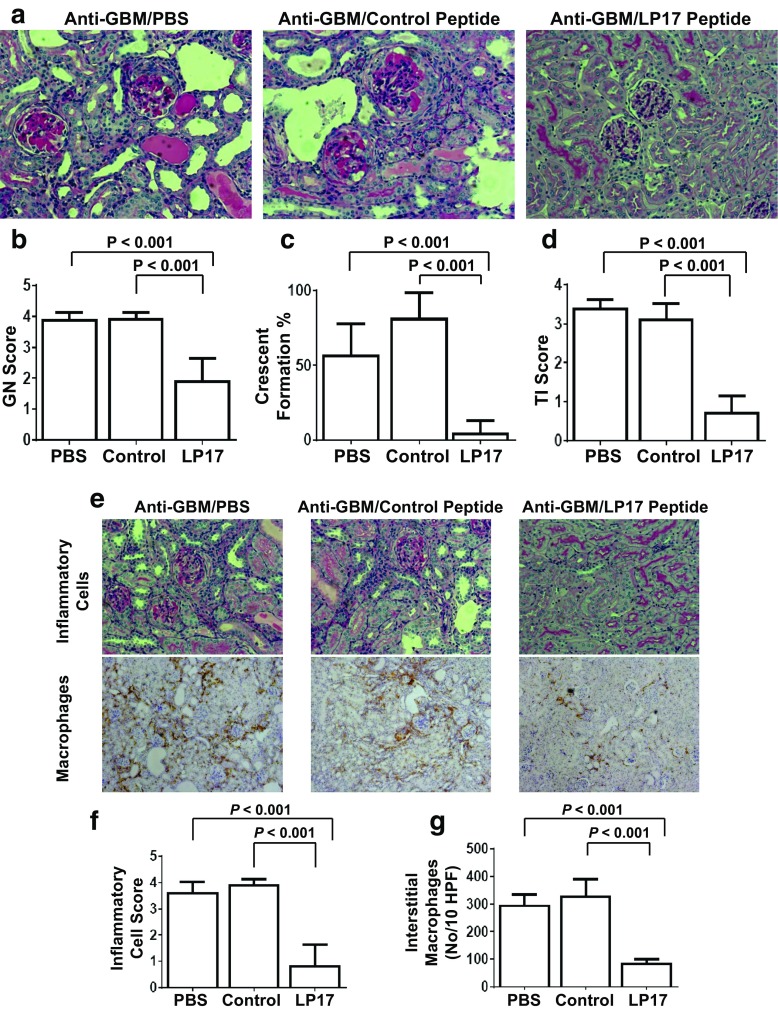
The LP17 inhibitory peptide dampens anti-GBM nephritis in 129/SvJ mice. Anti-GBM-induced mice were randomly divided into three treatment groups (*n* = 5) as in Fig. 1e, f. **a** An example of tissues from the three treatment groups is shown. Tissues were examined terminally for **b** glomerulonephritis (*GN*) score, **c** percent crescent formation, and **d** tubulointerstitial nephritis (*TI*) score. **e** Examples of tissues prepared from the three treatment groups examined for inflammatory cells (*upper panel*) or macrophages (*lower panel*). **f** Inflammatory cell infiltrate scores and **g** interstitial macrophage counts are shown at day 21 after receiving anti-GBM serum. Statistical analysis was carried out by Mann-Whitney *U* tests to obtain the *P* values shown.

Consistent with reported findings, 129/SvJ kidneys developed severe proliferative glomerulonephritis (GN) including mesangial proliferation with increases in matrices, focal necrosis, destruction of capillary lumens, and crescent formation [12, 13, 25, 28]. Figure 2 shows that renal disease, including GN score, percent crescent formation, and severity of tubulointerstitial (TI) disease, was markedly reduced in LP17 treated mice, commensurate with the dramatic reduction in the renal inflammatory infiltrate and interstitial macrophages in LP17-treated mice.

### Correlation of TREM-1 Expression with Renal Pathology

**Fig. 3:**
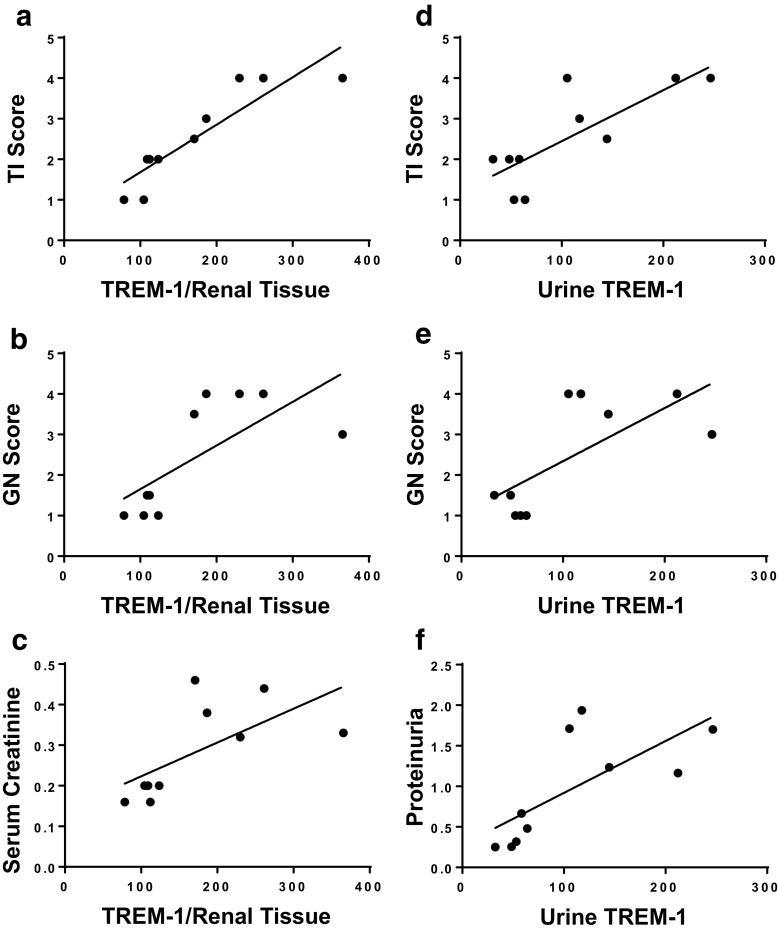
Correlation of TREM-1 expression with renal pathology. TREM-1 protein was detected in renal eluates from 129/SvJ and control B6 mice and correlated with **a** TI score (*r* = 0.8, *P* = 0.0004), **b** GN score (*r* = 0.5, *P* = 0.02), and **c** serum creatinine (*r* = 0.4, *P* = 0.009) as described in Fig. 2. Urinary soluble TREM-1 levels were also correlated with **d** TI score (*r* = 0.6, *P* = 0.006), **e** GN score (*r* = 0.5, *P* = 0.02), and **f** proteinuria (*r* = 0.5, *P* = 0.02) as described in “MATERIALS AND METHODS.” Statistical analysis was carried out by linear regression to obtain the *r* and *P* values.

Finally, we correlated the TREM-1 expression in anti-GBM-induced B6 and 129/SvJ kidneys with renal pathology scores as shown in Fig. 3. TREM-1 eluted from the kidneys of anti-GBM-diseased mice with varying disease severity correlated with tubulointerstitial disease (TI) score, glomerulonephritis (GN) score, and serum creatinine levels (Fig. 3a–c). Similarly, there was a clear correlation between renal pathology and urine soluble TREM-1 levels (Fig. 3d–f). Thus, these studies demonstrate that elevated levels of TREM-1 can be observed in anti-GBM-mediated nephritis and correlate with the degree of renal disease.

## DISCUSSION

Initial reports showed that monocytes, macrophages, and neutrophils expressed the TREM-1 receptor and that it was a potent costimulator of proinflammatory cytokines [14–16]. More recent studies have found that TREM-1 mRNA and protein were elevated in inflammatory bowel disease, experimental colitis, inflammatory arthritis, and other noninfectious inflammatory diseases [14, 21, 22]. Moreover, TREM-1 activation in the absence of TLR ligation or other stimuli induced myeloid cell cytokine production including TNF, IL-6, IL-8, and MCP-1 in *in vitro* cultures and disease models [14, 20, 21].

The current studies demonstrate that anti-GBM nephritis is associated with increased expression of TREM-1 (CD354) protein and mRNA in renal tissue from the 129x1/SvJ mouse strain in contrast to the nephritis resistant B6 strain. Urine levels of soluble TREM-1 but not serum soluble TREM-1 increased significantly in samples from the disease-susceptible 129x1/SvJ nephritic strain, indicating that the local production of TREM-1 in the inflamed kidney might contribute to disease pathogenesis. Although soluble TREM-1 and TREM-2 have been shown to attenuate or restrain macrophage activation, our data suggest that TREM-1 and TREM-2 expression levels are in part dependent on genetic influences and that increased TREM-1 expression promotes the inflammatory state. Our immunohistochemistry studies in murine and human lupus renal samples indicate that TREM-1 can be expressed by the inflammatory infiltrate and renal epithelial cells in chronic disease (manuscript in preparation). Thus, collectively, our data suggest that genetics could influence the relative expression of TREM-1 by the inflamed target organ.

Our studies are the first to demonstrate the elevated expression of renal TREM-1 in the nephritis-prone 129/SvJ strain and indicate that TREM-1 plays a critical role in the pathogenesis of inflammatory nephritis. Moreover, these studies demonstrate, for the first time, that TREM-1 is amenable to therapeutic targeting for nephritis. TREM-1 blockade markedly reduced inflammation in immune-mediated nephritis. These studies suggest that TREM-1 blockade might represent an effective novel strategy to be incorporated into induction and possibly maintenance regimens for patients with immune-mediated nephritis.
